# Doppler Assessment of the Fetal Brain Circulation

**DOI:** 10.3390/diagnostics16020214

**Published:** 2026-01-09

**Authors:** Maria Isabel Sá, Miriam Illa, Luís Guedes-Martins

**Affiliations:** 1 Instituto de Ciências Biomédicas Abel Salazar, University of Porto, 4050-313 Porto, Portugal; luis.guedes.martins@gmail.com; 2 Serviço de Obstetrícia, Departamento da Mulher e da Medicina Reprodutiva, Centro Materno Infantil do Norte, Unidade Local de Saúde de Santo António, Largo Prof. Abel Salazar, 4099-001 Porto, Portugal; 3 Institut de Recerca Sant Joan de Déu (IRSJD), Fundació Sant Joan de Déu, 08950 Esplugues de Llobregat, Barcelona, Spain; miriam.illa@medicinafetalbarcelona.org; 4 Spanish Network in Maternal, Neonatal, Child and Developmental Health Research (RICORS-SAMID, RD24/0013/0004), Instituto de Salud Carlos III, 28040 Madrid, Spain; 5 Unidade de Investigação e Formação, Centro Materno Infantil do Norte, 4099-001 Porto, Portugal; 6 Instituto de Investigação e Inovação em Saúde, Universidade do Porto, 4200-319 Porto, Portugal

**Keywords:** Doppler ultrasound, fetal cerebral circulation, brain malformations, anemia, placental vascular anastomoses, monochorionic diamniotic twins, neurodevelopment

## Abstract

Doppler assessment of fetal cerebral circulation has become a cornerstone of modern fetal medicine. It is used to evaluate cerebral vascular malformations, brain anomalies, fetal growth restriction due to placental insufficiency, fetal anemia, and hemodynamic complications arising from placental vascular anastomoses in monochorionic pregnancies. Emerging research also explores the predictive value of Doppler parameters for perinatal outcomes and long-term neurodevelopment. To review the anatomy and physiology of fetal cerebral vessels accessible to Doppler evaluation, outline key technical aspects, and summarize current obstetric applications. A PubMed search identified 113 relevant publications, published between 1984 and 2025. Three book chapters by authors recognized internationally within the scientific community were included. A total of 116 publications were critically analyzed in this narrative review. Strong evidence supports the use of Doppler ultrasound in obstetrics, particularly for evaluating fetal cerebral hemodynamics, where it contributes to reducing fetal morbidity and mortality. Doppler assessment of fetal brain circulation is a valuable tool for evaluating brain vascular malformations, other structural abnormalities, and for assessing fetuses with growth restriction, anemia, and twin-to-twin transfusion syndrome. It allows targeted fetal monitoring and timely interventions, providing critical prognostic information and aiding parental counseling. Ongoing advances in Doppler technology and understanding of fetal brain physiology are likely to broaden its clinical uses, improving both perinatal outcomes and long-term neurological health.

## 1. Introduction

Since Ian McDonald first visualized the fetal head in 1959 using ultrasounds, imaging technology applied to obstetric ultrasound and Doppler has undergone extraordinary advancements. These developments have revolutionized pregnancy management and paved the way for the rapid evolution of Fetal Medicine as a distinct discipline. Beyond elucidating the physiology and normal trajectory of fetal development, including fetal brain and its vascularization, these technologies have enabled the precise identification of deviations from normality and a deeper understanding of the natural history of numerous fetal pathologies. Their contribution is indisputable, as conditions that once remained undetected in utero and were often associated with adverse outcomes can now be diagnosed, monitored, and, in many cases, treated before birth. This progress has fundamentally altered clinical decision-making, allowing for more effective surveillance, timely intervention, and optimized delivery planning, ultimately improving perinatal prognosis and neonatal outcomes [1,2,3,4,5,6,7,8,9,10,11,12,13,14].

In the 1970s, the first hemodynamic indices were described, along with the flow patterns of fetal vessels [1,2,15,16,17]. Over subsequent decades, several groups published research on the anatomy of fetal brain vessels observed through ultrasound, based on anatomical knowledge from postmortem fetal studies; explored the technical aspects of Doppler evaluation and the methods for measuring flow parameters; and developed reference curves for flow indices of fetal cerebral vessels [1,2,18,19,20,21,22,23,24,25,26,27,28].

Fetal brain Doppler soon began to be used in various aspects of fetal medicine, from cerebral vascular malformations [29,30,31,32,33] to the diagnosis of brain anomalies [34,35,36,37,38,39,40,41].

Currently, the assessment of fetuses with growth restriction secondary to placental insufficiency represents one of the most established and clinically relevant applications of fetal cerebral Doppler in obstetrics [42,43,44,45,46,47,48,49,50,51,52]. However, the evaluation of fetal anemia and the investigation of hemodynamic complications resulting from placental vascular anastomoses in monochorionic pregnancies also constitute frequent and valuable indications for its use [53,54,55,56,57,58,59,60,61,62,63,64,65,66,67,68,69,70,71,72,73]. An additional and increasingly active field of research concerns the potential of Doppler parameters to predict not only perinatal outcomes but also long-term neurodevelopmental trajectories [74,75,76,77,78,79,80,81,82,83,84,85,86,87,88,89,90,91,92,93].

In this text, we aim to explore the current applications of fetal brain Doppler assessment in Obstetrics, briefly reviewing the technical issues inherent to this technology, the anatomy of the cerebral vessels accessible to Doppler study, and their physiology.

## 2. Materials and Methods

A comprehensive search was conducted in the PubMed database using the MeSH terms “fetal development,” “cerebrovascular circulation,” “autoregulation,” “ultrasonography, Doppler,” “ultrasonography, Doppler color,” “cerebral arteries,” “neuroanatomy,” “blood flow velocity,” “congenital abnormalities,” “arteriovenous malformations,” “fetal growth retardation,” “pregnancy complications,” “anemia,” “fetofetal transfusion,” “fetal mortality,” “neurodevelopment disorders,” “artificial intelligence,” and “mentoring.” Each study was evaluated for relevance to the narrative review of fetal cerebral Doppler assessment.

Publications were screened for eligibility by a single author (M.I.S.) according to predefined inclusion criteria. Eligible works comprised articles, books, clinical guidelines, and opinion papers authored by recognized experts, provided that an abstract was available in English and the publication date ranged from 1984 to 2025.

**Figure 1:**
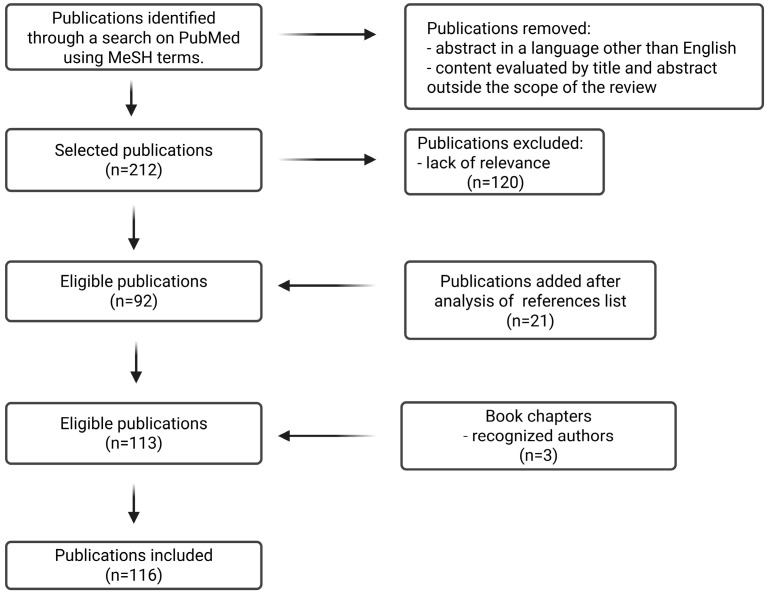
Search results flowchart.

Following title and abstract screening, 212 articles were initially selected as relevant to the objective of the narrative review. Reference lists of the included studies were also examined, resulting in an additional 21 articles. Three book chapters by internationally recognized authors within the scientific community were included. After full-text review, 120 studies were excluded due to lack of relevance. In total, 116 publications are included in this manuscript, which focuses on the current state of Doppler evaluation of fetal cerebral circulation and its clinical applications (Figure 1).

## 3. Fetal Cerebral Vascular Maturation

### 3.1. Development of the Fetal Cerebral Arterial Tree

In the last fifty years, fetal brain circulation became an important area of investigation. Several works, the oldest dating back to the 1970s, brought to our attention that the fetal cerebrovascular balance differs significantly from the adult and imbalance consequences are markedly dependent on gestational age [3,4].

Animal studies were the true starting point for understanding the structure, physiology, maturation and regulation of fetal cerebral vasculature [3,4]. Animal studies, primarily conducted in rodents, have demonstrated the need for caution when extrapolating findings to humans. This is due, among other factors, to differences in the timing of brain development between species. For instance, the stage of brain maturation in rodents at birth corresponds approximately to that of the human fetus at 16 weeks of gestation, while the rodent brain at postnatal day 14 (P14) resembles that of the human fetus at around 29 weeks of gestational age [4].

From an anatomical point of view, at four weeks of gestation, the development of the internal carotid arteries can already be observed. The proximal portion of these vessels results from the developing third aortic arch. Anastomoses between the first six dorsal intersegmental arteries give rise to the vertebral arteries posteriorly, which then fuse to form the basilar artery. The development of the anterior and middle cerebral arteries (originating from the internal carotid artery), the posterior cerebral arteries (arising from the basilar artery), and the anterior and posterior communicating arteries completes the formation of the Circle of Willis by approximately seven weeks of gestation [5,6,7].

Animals studies documented that from a structural point of view, in general, fetal arteries have smaller smooth muscle cells, significantly larger extracellular space with larger amount of water than adult cerebral arteries do [3]. Also, fetal cerebral arteries seem to be more compliant and more reactive both to stretch and to aminergic agonists probably due to a higher sensitivity to calcium [3]. There is also evidence that reactivity to nitric oxide is higher in immature than mature cerebral vessels, and in the same way, the synthesis and levels of cGMP are higher in neonates than in adults [3]. Adrenergic, cholinergic, and serotonergic vascular neuroeffector mechanisms appear to differ significantly between fetal and adult arteries. In particular, serotonin and norepinephrine have been shown to induce vasoconstriction in fetal cerebral arteries [3]. Additional animal studies have demonstrated a reduced response of fetal arteries to external electrical stimulation, suggesting immature sympathetic perivascular innervation during fetal life [3]. Moreover, fetal cerebral arteries appear to undergo structural and functional maturation later than carotid arteries [3]. These vessels also exhibit greater compliance and heightened reactivity to both stretch and aminergic agonists, likely reflecting increased calcium sensitivity [3]. Furthermore, evidence indicates that reactivity to nitric oxide is greater in immature cerebral vessels compared to mature ones. Correspondingly, both the synthesis and concentration of cyclic guanosine monophosphate (cGMP) are higher in neonates than in adults [3]. Wagner et al. also demonstrated the existence of progesterone receptors in the fetal rat brain, as well as the correlation between serum levels of this hormone in fetal and maternal serum, suggesting the role of this hormone in the activation of nuclear receptors and subsequently in the production of transcription factors involved in mechanisms essential to neurological maturation [8].

### 3.2. The Neurovascular Unit and Its Functions

**Figure 2:**
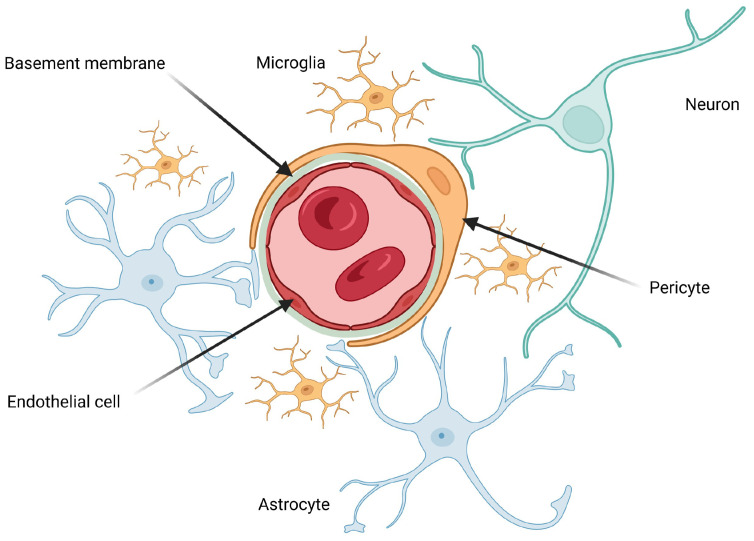
Neurovascular Unit: blood vessel surrounded by the basement membrane and closely related to the “end-feet” processes of perivascular astrocytes, pericytes, neurons, and microglia. Multiple signaling pathways enable interactions between these elements, ensuring the integrity of the blood–brain barrier and the autoregulation of blood flow in response to the metabolic needs of the fetal brain. Created in BioRender. Sá, M. (2026) https://BioRender.com/6or6ion.

In 2001, a new concept in Neuroscience emerged, the Neurovascular Unit, that represented the anatomical and functional relationship between de cellular components of the brain (neurons, perivascular astrocytes, microglia, pericytes, endothelial cells, and basement membrane) and blood vessels (Figure 2) [4,9]. Since then, research on Neurovascular Unit quickly grew and there is now a significant knowledge on this subject, especially concerning the adult brain [4].

One of the main functions of the Neurovascular Unit is the barrier function. Endothelial cells play a major role providing an adequate environment for brain functioning.

Fetal brain endothelial cells have characteristics that distinguish them from other endothelial cells. They are associated with a thinner wall, the absence of fenestrations, the presence of tight junctions and significant differences between the luminal and anti-luminal membrane, with respect to expression of enzymes, receptors, ion channels and transporters, resulting in a highly selective barrier that allows the fetal brain to capture the substances necessary for its metabolism and excrete the resulting products [4,9,10].

However, other cells like astrocyte, pericyte and neurons, might participate in this environment balance [4,9]. Astrocytes are found between the vessels and neurons, and have “end-feet” processes that extend in the vessels, acting as mechanic elements. Furthermore, they have a high concentration of aquaporin 4 water channels and potassium transporters which enables them to control ions, neurotransmitters and water balance [4,9]. Pericytes also play a major role in maintenance of blood–brain barrier. They influence gene expression leading to increase in production of endothelial-cell products that favour barrier function and mediate the attachment of astrocyte end-feet to vessel walls [4].

There is evidence that pericytes appear in the fetal brain at a very early stage of development, forming primary vascular microstructures and subsequently recruiting endothelial cells by producing vascular endothelial growth factor [4]. On the contrary, astrocytes appear at a later stage, with “end-feet” processes reaching close contact with the vessels in the second half of gestation [4].

In functional terms, contrary to what was thought in the past, the blood–brain barrier in the fetus is already functioning before birth, but its development occurs at different points during gestation for different brain areas with decrease in permeability with gestational age [4].

The basement membrane, despite not having any direct barrier function, being composed of a matrix of proteins produced by pericytes and endothelial cells, contributes to vascular integrity by helping to support the surrounding vessels and cells [4,9].

The second major function of the Neurovascular Unit is the neurovascular coupling, which refers to the capacity of changing blood supply by vasoconstriction/vasodilation depending on tissue metabolic demands [4,9].

Neurons are highly sensitive to fluctuations in nutrient and oxygen levels. In response, they release glutamate, which activates adjacent astrocytes and pericytes, triggering the release of vasoactive molecules [4,9]. The complexity of cerebral blood flow regulation in the fetal brain is exemplified by the role of astrocytes. These cells respond to synaptically released glutamate by increasing intracellular free calcium concentrations, which in turn promotes the release of vasodilatory arachidonic acid metabolites [4,9]. However, evidence suggests that this same increase in intracellular calcium can also lead to vasoconstriction, depending on factors such as tissue oxygen tension, the magnitude of the calcium rise, basal vascular tone, and the concurrent activity of nitric oxide [4]. Pericytes, which are intimately associated with endothelial cells, are central to the regulation of vessel diameter. They express receptors for various vasoactive substances and contain contractile proteins; upon activation, these proteins modulate pericyte contractility, leading to either vasodilation or vasoconstriction depending on the context [4]. Evidence is still very limited regarding the role of endothelial cells in regulating fetal cerebral vascular flow; however, it is known that they produce vasoactive substances [4].

Neurons also play a major role in brain development and organization stimulating angiogenesis. There is evidence that insults to fetal neurons lead to potential irreversible stop in angiogenesis even if the insult is withdrawn conditioning later brain development [4].

### 3.3. Impact of Hypoxia and Growth Restriction on Fetal Cerebral Circulation

The differences in response to insults also occur between fetal and adult brains. Researchers have focused primarily on the effects of three frequent insults to the fetal brain: prematurity, acute hypoxia, and chronic hypoxia.

One of the major causes of neurovascular unit disfunction induced by prematurity is germinal matrix hemorrhage that causes intraventricular hemorrhage leading potentially to cerebral palsy, post-hemorrhagic hydrocephalus, and severe cognitive impairment [4]. This occurs mainly in infants under 32 weeks of gestational age because blood–brain barrier in germinal matrix by this point has specific characteristics, different from other brain areas, that make vessels vulnerable to changes in blood pressure and other insults. These include underdevelopment of the basement membrane and endothelial tight junctions, lack of expression of some proteins in the end-feet processes of astrocytes and reduced pericyte coverage [4]. It should be noted that there is evidence that these characteristics that put the germinal matrix at risk below 32 weeks appear to be protective against other types of damage, such as inadequate inflammatory responses in some contexts that occur in full-term fetuses and not in preterm fetuses [4].

Although the high incidence of intraventricular hemorrhage in preterm infants and the high vulnerability of preterm fetus to hypoxia were well documented long before, it was only with the beginning of investigation in animal models that some light was brought to the mechanisms of fetal cerebral vascular regulation in response to ischemia. These models showed how heterogeneous the distribution of blood flow was in different brain areas, favouring distribution through the brain stem, with optimal correlation between metabolism and perfusion [3,11]. They also stablished the association between decreased oxygen levels/increase carbon dioxide levels and fetal cerebral vasodilation [11]. It was demonstrated, using lamb models, that there was a significant increase in fetal cerebral flow following a decrease in oxygen levels, being the mean response 40% higher for the brainstem than for the cortical and subcortical areas [11]. In the same way, cerebral flow increased with elevation of carbon dioxide content, also favouring the brainstem [9]. On the other hand, it seems that cerebral blood flow remains stable in a quite wide range of values for cardiac output and blood pressure [11].

Studies observing the pial arteries in animal models also documented the sympathetic innervation and the contractility capacity of the fetal brain vessels in response to prostanoids [3]. It became known that the vasodilator response decreases with gestational age while the vasoconstrictor response increases with gestational age [3].

It is now widely accepted that hypoxia—whether due to hypoxemia or ischemia—initiates a cascade of complex pathophysiological events which, if sufficiently severe, may culminate in hypoxic–ischemic encephalopathy. The first phase of this cascade is known as primary energy failure, corresponding to an abrupt reduction in cerebral blood flow. There is robust evidence that this stage is associated with increased permeability of the blood–brain barrier, alterations in the expression of tight junction proteins, ion and amino acid transporters and pumps, as well as disruption of cytoskeletal structure and vascular cellular integrity [4]. This initial insult is followed by a latent phase lasting approximately 6 to 24 h, during which cerebral perfusion is progressively restored. During this period, changes occur in the expression of transcription factors and inflammatory cytokines [4]. The subsequent phase, referred to as secondary energy failure, is characterized by mechanisms that exacerbate neuronal and glial injury, contributing to the progression of tissue damage [4]. This damage seems to be related with the inflammatory response to ischemia, mediated by chemokines, cytokines, reactive oxygen species, secondary messengers and matrix metalloproteinases, all coordinated mainly by microglia that increase significantly after a hypoxic insult [4]. Astrocyte number and size also increase significantly with acute hypoxia [9].

One of the intervenients in cerebral effects of hypoxia in fetal life appears to be ornithine decarboxylase [3]. Its levels increase in fetal brain tissue when oxygen acutely drops, and the same happens when carbon dioxide is administered to the mother [3]. These effects seem to be mediated by oxygen radicals [3].

These findings are also supported by animal models that demonstrate the association between fetal asphyxia and vasogenic brain oedema and the insufficiency of the fetal cerebral autoregulation in response to hypoxia1with vasodilation persisting for periods much longer than the hypoxia itself [3].

Contrary to acute hypoxia, chronic hypoxia is characterized by marginal reductions in oxygen levels which are persistent and might last for several weeks or even months. While acute hypoxia in fetal life results in arterial vasodilation based on endothelium-independent mechanisms, chronic hypoxia showed to induce structural and functional changes that differ from the response in adult brain, being the fetal brain more susceptible to damage [4].

There is evidence that, during fetal life, chronic hypoxia induces a redistribution of blood flow that preferentially favours vital organs such as the brain, heart, and adrenal glands. This adaptive response is reflected in a phenomenon of cerebral vasodilation known as brain sparing. However, this neuroprotective mechanism is only partially effective. It has been associated with several adverse neurodevelopmental outcomes, including a reduction in the number of fetal brain cells, particularly glial cells such as astrocytes and oligodendrocytes, decreased myelination, reduced cortical gray matter and total brain volume at birth, impaired angiogenesis, diminished responsiveness of cerebral arteries to vasoactive stimuli, and alterations in both perivascular innervation and endothelial cell function. Additionally, pericyte hypertrophy contributes to increased vascular wall thickness and reduced pericyte coverage in certain brain regions, such as the germinal matrix. These structural and functional changes are further exacerbated by the release of pro-inflammatory cytokines from activated glial cells, including microglia and astrocytes [4,9,12,13]. This changes result in reduction in cerebrovascular contractility and anomalies of the mechanisms of neurovascular coupling, a higher risk of hemorrhage in some brain areas like the germinal matrix and anomalies of the blood–brain barrier with an increase in permeability. Animal studies in growth restricted piglets demonstrated that the increase in blood–brain barrier permeability leads to immune T cells migration to perivascular regions and brain parenchyma, being this neuroinflammation potentially harmful depending on the subtypes of T cells involved [9]. Interestingly, changes related to chronic hypoxia appear to increase the risk of intraventricular hemorrhage mainly in fetuses with higher gestational age near term [4]. Studies in growth restricted lambs, demonstrated that microglia, involved in neuroinflammation, was observed only at 124 days gestational age suggesting that the period of perinatal development when the neurovascular unit is most vulnerable to neuroinflammation and disruption is around this gestational age, which is equivalent to 34 weeks in humans. This supports the data from human infants, that show that preterm growth restricted infants born after 34 weeks have higher risk of intraventricular hemorrhage than growth restricted infants born at 28 weeks [9].

At the same time that all these harmful changes occur due to insults such as prematurity and acute or chronic hypoxia, there is evidence that there are processes of preservation and regeneration of brain tissue triggered by these same insults. Astrocytes might play an important role following ischemic injury leading to changes in metabolic pathways and mitochondrial membrane function to improve neuronal survival. Also pericytes might be involved in restorative responses, interfering in angiogenesis, neurogenesis, and immune regulation. There is also some evidence that pericytes might share some features with mesenchymal stem cells, having a potential for regenerative processes. In the same way, animal models show a decrease in dendrite branches after a hypoxic insult that is followed by an increase, which may reflect a compensatory and adaptation mechanism [13].

Two treatment approaches have proven effective so far in reducing the risk of adverse neurological events linked to prematurity. Administration of corticosteroids (betamethasone or dexamethasone) is associated, in addition to decreasing the risk of respiratory distress syndrome, with a lower risk of intraventricular hemorrhage. Antenatal magnesium sulfate administration is also linked to a reduced risk of cerebral palsy, likely because it interferes with mechanisms that regulate cerebral vasodilation and decreases inflammatory cytokines, oxygen free radicals, and cellular calcium influx [14].

It is true that, in terms of understanding the development and maturation of the fetal brain, we are still at the early stages of a long and complex research journey. Nevertheless, it is already clear that fetal brain development involves multiple, dynamic, and highly intricate processes that vary according to gestational age. In the face of an insult, these processes interact in ways that can confer either vulnerability or resilience, depending on the timing of the event. As a result, the functional outcome for the brain may be more or less favourable, depending on the developmental stage at which the insult occurs.

Understanding the mechanisms of autoregulation of fetal cerebral vascularization, as well as the described pathophysiological phenomena, allows us to interpret and understand the evolution of hemodynamic indices used in the Doppler study of fetal cerebral vascularization, as we will see below.

## 4. Vascular Ultrasound Technology

### 4.1. Basic Doppler Principles

Ultrasonography has become an essential tool for studying the fetal brain, especially Doppler imaging.

To understand the mechanism of obtaining fetal images through this technique, sound must be viewed as mechanical energy propagating through a given medium in the form of pressure waves. The ultrasound probe contains piezoelectric materials, such as lead zirconate titanate, which, when applied to an electric current, vibrate, creating sound waves. These, in turn, undergo tissue reflection processes, returning to the probe, where the piezoelectric material converts the sound waves back into an electric current, generating anatomical images [15].

A sound wave can be described by several acoustic parameters, such as period (the duration of a complete cycle expressed in microseconds), frequency (the number of cycles per second expressed in megahertz), wavelength (the distance between two corresponding points of consecutive cycles expressed in millimeters), amplitude (the difference between the maximum and average values of the wave), power (the energy generated per unit of time expressed in watts), intensity (the amount of energy in the cross-section of the sound beam, expressed in watts per square centimeter), and propagation speed (the distance sound travels in one second in a given medium, expressed in meters per second) [15]. Naturally, these variables are related to each other in specific ways: period and wavelength are inversely proportional to frequency; intensity is directly proportional to power, and both are related to amplitude [15].

The speed of sound propagation depends only on the medium in which it travels. In the human body, the speed is highest in solids, lowest in gases, and intermediate in liquids. The two characteristics that define the propagation speed are stiffness and density, with stiffness increasing and density decreasing the speed [15].

Another significant tissue effect on sound is attenuation, which refers to the weakening of sound as depth increases. In water, there is negligible attenuation for commonly used frequencies, making this medium an ideal acoustic window. Attenuation is also greater at higher frequencies. Higher frequencies produce images with higher resolution, enabling the visualization of finer details; however, they have a reduced penetration depth. Low-frequency probes penetrate deeper, but the resulting image has lower resolution [15].

One potential clinically relevant cause of attenuation is absorption. Absorption refers to the conversion of acoustic energy into thermal energy in tissues; the greater the absorption, the greater the amount of heat generated [15].

After the probe receives the sound signal, it is converted into an electrical signal, and the data is collected and converted into an image. This image can be displayed in various ways. The amplitude mode (A-mode) is a basic ultrasound imaging method that focuses on analyzing echo amplitude as a function of depth. Electrical signals are represented as peaks on a graph. The height of each peak corresponds to the echo amplitude, showing the strength of the reflected signal. The horizontal position of the peak indicates the depth or distance from the structure that caused the echo. It is useful in specific applications that need precise distance measurements [15]. Brightness mode (B-mode) creates anatomical images from the different amplitudes received by the transducer, weaker signals appearing as darker dots and stronger signals appearing as brighter dots [15]. Motion mode (M-mode) detects tissue movement, representing fixed structures as straight lines, and moving structures as sinusoidal lines [15].

### 4.2. Doppler Effect, Color, Power and Spectral Doppler

In clinical practice, one of the primary uses of the Doppler effect is in blood flow studies. When blood particles approach the probe, they transmit sound waves of greater intensity than those generated by it (positive shift); when they move away, they transmit sound waves of lower intensity than those generated by the probe (negative shift). The velocity of blood flow determines the Doppler shift; higher velocity results in larger changes [15]. The shift also depends on the angle between the sound waves coming from the ultrasound probe and the vessel; there is no detectable shift at an angle of 90°, whereas the most significant possible shift occurs at an angle of 0° between the sound wave and the vessel [15]. Since the blood flow velocity is equal to the distance traveled over the analysis time, knowing the time interval between pulses, it is possible to determine several velocity parameters and thus characterize the hemodynamic state of a given vessel [16].

Currently, several Doppler modalities are utilized in ultrasound studies, including color Doppler, power Doppler, and spectral Doppler.

Color Doppler flow imaging is among the most used techniques. It utilizes Doppler shifts to display flow simultaneously with B-mode image acquisition, employing black and white coding to represent structures and color coding to represent flow velocity [16]. When color Doppler is turned on, a color scale appears on the ultrasound device monitor, usually in different intensities of blue and red, showing that the color at the top of the scale indicates direction toward the probe. In contrast, the color at the bottom of the scale indicates direction away from the probe [17]. The highest measurable velocity, beyond which the Doppler may inaccurately depict the velocity and direction of blood flow, is called the Nyquist limit. When blood flow velocity exceeds the limits of the color scale, blood flow is shown in the opposite direction, that is, with the opposite color. This phenomenon is called aliasing [17].

Power Doppler modality represents only the amplitude of frequency shifts detected by the probe, without analyzing velocity or direction. This permits the detection of slow flows [17].

**Figure 3:**
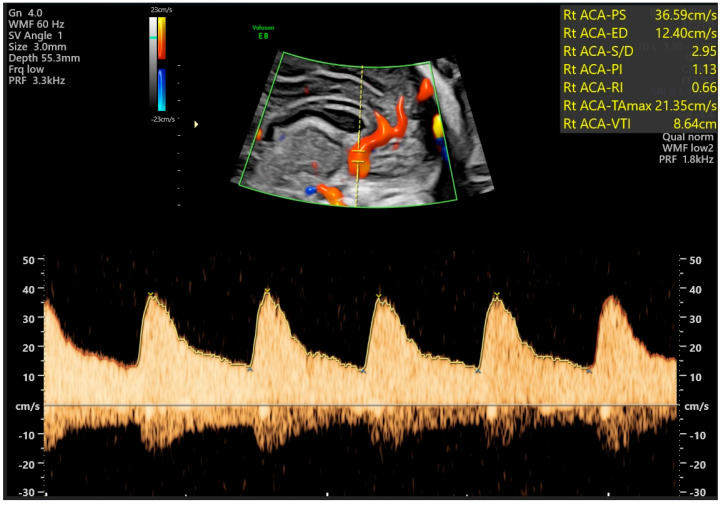
Doppler assessment of the anterior cerebral artery flow wave using pulsed-wave Doppler. Mode B displays structures in black and white, while Doppler shifts illustrate blood flow. The code in the top left indicates the expected color for flow approaching or moving away from the probe, with the top of the scale indicating direction toward the probe. In this case, the anterior cerebral artery appears red in relation to the corpus callosum. Spectral Doppler provides a graphical representation of the flow wave below, allowing analysis of velocity as a function of time. This analysis offers the operator several hemodynamic parameters at the top right, such as systolic and diastolic velocities, the ratio of systolic to diastolic velocities, the pulsatility index, the resistance index, the maximum velocities average, and the velocity-time integral.

Finally, spectral (or quantitative) Doppler returns to the operator a graphical representation of velocity (vertical axis) as a function of time (horizontal axis), with three modalities being possible: pulsed-wave, continuous-wave, and tissue Doppler. When using pulsed-wave Doppler, a sample gate of approximately the same size as the vessel diameter is placed in a given vascular segment at a predetermined depth. A single ultrasound probe crystal is used to both emit and receive ultrasound, emitting a new pulse only after having received the immediately previous one. This enables the graphical representation of blood flow in that vascular segment, thereby obtaining various hemodynamic parameters, such as systolic and diastolic velocities, for example (Figure 3) [17]. Unlike pulsed-wave Doppler, continuous-wave Doppler uses a system in which the probe simultaneously emits and receives the reflected sounds continuously, from the entire path being analysed, allowing for high-frequency wave detection. This allows for high-velocity flow analysis but with limited depth discrimination [17]. Finally, tissue Doppler applies the Doppler effect to the study of tissue structures in motion. An example of this modality’s applicability is the study of fetal myocardium [17].

## 5. Anatomic, Technical, and Methodological Considerations

This text does not seek to provide a comprehensive review of the anatomy of the cerebral vasculature, but rather to analyze the principal vessels, particularly those that can be assessed by ultrasound during the fetal period.

### 5.1. Overview of Fetal Cerebral Arteries Relevant to Doppler

The brain’s arterial blood supply mainly comes from branches of the carotid and subclavian arteries. The common carotid arteries travel through the deep cervical fascia and bifurcate into the external and internal carotid arteries at the level of the fourth cervical vertebra [18]. The internal carotid artery runs anteriorly to the transverse processes of the upper three cervical vertebrae, enters the cranium through the petrous part of the temporal bone, and supplies blood to the same side cerebral hemisphere, eye, forehead, nose, nasal cavity, and paranasal sinuses [18]. The subclavian arteries give rise to several branches, including the vertebral arteries. These vessels pass through the transverse foramen of the first six cervical vertebrae, enter the cranium through the foramen magnum, and fuse medially anterior to the pons to form the basilar artery [18]. They supply the upper spinal cord, brainstem, cerebellum, and occipital lobe of the brain [18].

**Figure 4:**
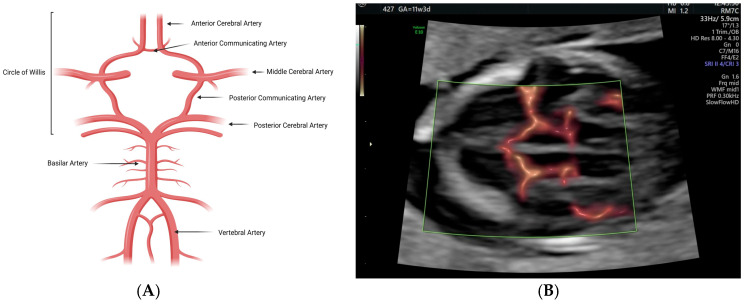
Circle of Willis. (**A**)—Diagram of the Circle of Willis. Note the anterior portion formed by the two anterior cerebral arteries and the anterior communicating artery, and the posterior portion formed by the posterior cerebral arteries and the two posterior communicating arteries. From the junction of the two portions, the two middle cerebral arteries emerge. Created in BioRender. Sá, M. (2026) https://BioRender.com/hrv3iw9. (**B**)—Circle of Willis observed in the first trimester of pregnancy using B-mode combined with Power Doppler (area marked in green). The red color represents the signal amplitude, not the flow direction, allowing for the detection of low-velocity flow. In the middle, the two middle cerebral arteries can be seen as the largest vessels; to their left are the two anterior cerebral arteries that communicate in the midline through the anterior communicating artery; to the right, the two posterior communicating arteries and the two posterior cerebral arteries.

The branches of the internal carotid artery and vertebral arteries anastomose in the circle of Willis, which, as mentioned above, is already formed in the second half of the first trimester, and gives rise to four types of arteries: the convolutions arteries, the central nuclei arteries, the ventricular arteries, and the base arteries (Figure 4) [18,19].

From these arteries, we will mention the convolution arteries: anterior cerebral artery (ACA), middle cerebral artery (MCA), posterior cerebral artery (PCA), and anterior choroidal artery [19].

The ACA is a branch of the internal carotid artery that runs anteriorly to the interhemispheric fissure, where it anastomoses with its contralateral counterpart via the anterior communicating artery. The ACA has five segments: A1 (pre-communicating segment), which originates from the internal carotid artery and extends to the anterior communicating artery; A2 (post-communicating segment), which courses around the rostrum of the corpus callosum, extending from the anterior communicating artery to the genu of the corpus callosum until the origin of the callosomarginal artery; A3 (precallosal segment), originating at the callosomarginal artery’s origin and going around the genu and the rostral part of the corpus callosum; A4 (supracallosal segment), which accompanies the body of the corpus callosum until the coronal suture; and A5 (postcallosal segment), continuing superiorly to the corpus callosum after passing the plane of the coronal suture. Some authors group segments A3, A4, and A5 into a single segment called the pericallosal artery. The ACA supplies blood to the frontal lobe, parietal lobe, corpus callosum, and internal capsule [20].

The MCA is also a branch of the internal carotid artery and runs along the entire length of the Sylvian fissure. The main MCA trunk can be divided into four segments: M1 (sphenoid or horizontal segment) extending horizontally along the sphenoid wing; M2 (insular segment) extending vertically along the operculum; M3 (opercular segment) extending horizontally in the Sylvian fissure; and M4 (cortical branches) extending on the surface of the cortex [17,19,20]. From an evolutionary perspective, the MCA is the most recent cerebral artery in phylogenetic terms. Its development is due to the more complex evolution of the cerebral cortex in “higher” species, which led to the recruitment of vessels from the existing lenticulostriate system to supply the neocortex. Thus, contrary to the classical perspective in which the MCA is seen as a primary branch of the internal carotid artery, from an evolutionary perspective, the MCA can be seen as a branch of the ACA and of lenticulostriate vessels. As such, a great anatomical variation can be observed among individuals. The MCA is responsible for the vascularization of a large part of the lateral surface of the frontal, parietal, and temporal lobes, as well as the basal ganglia and the internal capsule through its lenticulostriate branches [22].

The two PCAs, right and left, are formed by the bifurcation of the basilar artery around the pontomesencephalic connection, which in turn results from the fusion of the vertebral arteries, as previously mentioned. These two arteries, together with the two posterior communicating arteries, constitute the posterior portion of the circle of Willis. This, in turn, connects to the anterior portion, completing the circle through the communication between the latter two and the internal carotid arteries and ACAs. The PCA circles the brainstem, passing through the crural and ambiens cisterns and reaching the quadrigeminal cistern, to finally distribute to the posterior part of the hemisphere. The PCA is formed by four segments: P1 (pre-communicating segment) extends from the basilar artery to the posterior communicating artery; P2 (post-communicating segment) from the posterior communicating artery to the lateral portion of the midbrain; P3 (quadrigeminal segment) from the posterolateral portion of the midbrain and ambiens cistern to the quadrigeminal cistern, till the anterior limit of the calcarine fissure; P4 (cortical-calcarine segment) that is composed by PCA major terminal branches, the calcarine and parieto-occipital arteries. In general, the PCA provides vascularization of the posterior and inferior surfaces of both cerebral hemispheres, including the occipital lobe and the inferior portion of the temporal lobe, but also sends critical branches to the diencephalon, mesencephalon, and ventricular system [19,23].

The anterior choroidal artery is a small-caliber vessel that originates from the internal carotid artery, usually 2–5 mm distal to the posterior communicating artery. It is divided into two segments: the cisternal segment, from its origin to the choroid fissure, and the intraventricular segment, which extends through the choroid plexus in the lateral ventricle until it gets PCA branches. Despite its small size, the anterior choroidal artery supplies blood to vital brain regions, including the posterior limb of the internal capsule, optic tract, lateral geniculate body, medial temporal lobe, and medial area of the globus pallidus [24].

### 5.2. Venous Structures Relevant to Fetal Doppler

Cerebral veins are divided into three types: the convolutional veins; the deep veins or veins of Galen; and the basal veins.

Regarding the convolutional veins, the internal veins, that is, those originating from the internal surface of the hemispheres, mostly drain ascendingly into the superior longitudinal sinus. The external cerebral veins, originating from the external surface of the hemispheres, are divided into ascending and descending veins. The ascending veins drain into the superior longitudinal sinus; the posterior descending veins drain into the lateral sinus; the anterior descending veins drain into the superior petrosal sinus or the cavernous sinus. The anterior inferior cerebral veins drain into the superior longitudinal sinus or the basal veins; the posterior inferior veins drain into the lateral sinus, the superior petrosal sinus, or the basal veins [19].

The deep veins or veins of Galen are two, right and left, and are intended for draining venous blood from the central nuclei, the oval center, and the ventricular walls. They result from the confluence of the septal veins (coming from the septum pellucidum and corpus callosum), the vein of the corpus striatum, and the vein of the choroid plexuses. They run posteriorly through the thickness of the tela choroidea and join at its base to form the Vein of Galen, which, after a course of 8 to 10 mm, drains into the anterior portion of the straight sinus [19].

The two basilar veins, right and left, are the continuation of the anterior cerebral veins that accompany the arteries of the same name up to the genu of the corpus callosum. From this point, they flow posteriorly to drain into the Vein of Galen and subsequently into the straight sinus. The two basilar veins communicate through two anastomoses: the anterior communicating vein in front of the optic chiasm, and the posterior communicating vein at the level of the anterior border of the protuberance. There is also a venous polygon that closely parallels the Circle of Willis, as well as multiple smaller-caliber venous anastomoses [19].

### 5.3. Practical Ultrasound Acquisition of Cerebral Vessels

Nowadays, most ultrasound scanners, using abdominal and vaginal probes, the Doppler modalities already mentioned and also 3D reconstruction modalities, can map most of the vessels previously described [25]. There are ultrasound devices with multiple different features, but they usually share common basic functions for image acquisition and optimization, as well as for performing simple measurements or more complex hemodynamic assessments.

One of these main functions is depth control. Depth should be set to the minimum necessary to visualize the intended structure; otherwise, increasing depth will decrease the overall resolution of the image. The image should be enlarged so that the object of study occupies most of the monitor.

Another function is gain control, which involves adjusting the intensity of echoes returning to the probe and, consequently, changing the echogenicity of the image on the screen. Increasing the gain makes the image more hyperechoic, while decreasing it makes the image less hyperechoic. One way to optimize the image (the gain) and reduce the effects of ultrasound attenuation by tissues is to use the Time Gain Compensation function, which allows the operator to adjust gain at different depths. Additionally, with the “focus” command, the focal zone of the ultrasound beam—the region where the sound beam is thinnest and most concentrated—can be adjusted, providing the best image resolution and improving the ability to distinguish between closely situated structures, thereby enhancing image quality at a specific depth [17].

Regarding the application of Doppler technology, specifically color Doppler and spectral Doppler, lower frequencies are preferred to measure higher flow velocities. The pulse repetition frequency (PRF), which is the number of pulses per second, must be adjusted according to the type of vessel being studied. Pulses all have the same duration, so for deeper images, only the returning time of the echoes to the probe varies. Decreasing PRFs increases the time for receiving ultrasound echoes, allowing for the analysis of signals that require longer listening times, such as those from deeper structures and slow-flow vessels [15].

Another ultrasound parameter that must be adjusted based on the vessel being examined is the wall motion filter (WMF). This filter removes low-frequency signals caused by tissue movement, especially from the vessel walls, allowing the Doppler spectral analysis to focus solely on blood flow. However, to evaluate areas with low-velocity flow, such as venous regions, the WMF should be set as low as possible to avoid blocking the detection of true low-frequency slow flow [26].

Also, since the Doppler shift is directly proportional to the blood velocity and the cosine of the angle between the vessel under study and the ultrasound beam, and no shift can be recorded at an incidence perpendicular to the flow, whereas the greatest shift occurs when the beam is parallel to the flow, when acquiring the B-mode image, care must be taken to ensure that the vessel under study appears on the monitor as parallel as possible to the beam emitted by the probe [15,17].

When using spectral Doppler, the target vessel image should be enlarged so that it fills most of the ultrasound monitor. A gate with dimensions roughly equal to the vessel’s diameter should be placed over the image of the vessel lumen to obtain the flow wave spectrum, with the insonation beam aligned as closely as possible with the flow direction. The larger the insonation angle, the greater the error in calculating flow velocity; errors of 2% and 6% occur at angles of 10° and 20°, respectively. In practice, the insonation angle should not exceed 30–60°, but if the goal of the Doppler study is to evaluate the velocity parameter (as in Doppler study of the MCA in fetal anemia cases, as we will see below), every effort should be made to achieve an angle of 0°. If the insonation angle is larger, an appropriate correction can be made using the “angle” command; ideally, this should be noted in the report.

Several hemodynamic indices have been described, and their use aims to quantify in numerical terms the behavior of the flow wave in a given vascular territory. The two most used indices in fetal assessment are based on the measurement of the Doppler shift throughout the fetal cardiac cycle and are the Resistance Index (RI), also called Pourcelot’s index (systolic peak − end diastolic flow/end diastolic flow), and the Pulsatility Index (PI) (systolic peak − end diastolic flow/temporal average frequency over one cardiac cycle). The temporal average frequency corresponds in practice to the average height of the flow wave. In general, a high PI is obtained when evaluating vascular territories of high resistance, while a low PI is obtained in the evaluation of vascular territories of low resistance. The PI should be preferred over the RI, as it considers the flow wave behavior throughout the entire cardiac cycle, which results in different values, for instance, when there is no end-diastolic flow. It is therefore superior, especially in studying high-impedance territories [27].

Physiologically, the PI varies among different vascular territories and depends on gestational age. This index is higher in the MCA than in the carotid arteries, ACA, or PCA, and in these vessels, the PI decreases with gestational age, while flow velocity increases [27]. Reference curves for the various published hemodynamic indices for each vessel should be consulted to ensure proper analysis of the flow waves.

### 5.4. Safety Considerations for Fetal Brain Doppler

As mentioned earlier, acoustic energy can be converted into thermal energy in tissues through absorption, and the greater the absorption, the more heat is generated. Furthermore, sound waves are also a source of mechanical energy when they propagate through tissues. Another concern with the use of ultrasound is the phenomenon of cavitation. This occurs when the emitted waves interact with microscopic gas bubbles. Exceeding the peak rarefactional pressure amplitude threshold, the gas expands explosively to two or more times its initial diameter and then collapses. This can lead to damage to nearby tissues, either through mechanical and thermal processes or the release of free radicals. Therefore, all these effects could pose theoretical risks to the patient, especially to the fetus, as tissues and organs are in a state of rapid development. To ensure safety in the clinical use of ultrasound, thermal and mechanical indices were developed, which are part of the ultrasound device information [28]. Thermal index (TI) reflects the maximum potential temperature increase in tissues for the acoustic output of each device, considering prolonged tissue exposure. This information is available on the ultrasound device’s monitor in real-time and can be specified for the following thermal index types: soft tissue TI, bone TI, and cranium TI. From a practical standpoint, in terms of actual risk, TI values below 0.7 are considered risk-free, regardless of the duration of exposure. Conversely, TI values above 6 pose a risk of tissue damage for more than one minute of exposure, making it inadvisable. The mechanical index (MI), which reflects the risk of cavitation, is also displayed to the operator on the ultrasound screen. The maximum safe output limit for the ultrasound devices used is 1.9 [28].

To date, no harmful effects have been documented from the use of obstetric ultrasound for screening and diagnostic purposes. On the other hand, it has not been proven that the use of ultrasound is completely free of biological risk. Therefore, it is recommended that the ALARA (As Low As Reasonably Achievable) principle be applied. This means that the operator must ensure that acoustic exposure and examination duration are kept to a minimum to obtain the images necessary for a correct diagnosis [28].

## 6. Brain Vascular Malformations in the Fetus

### 6.1. General Considerations

Congenital cerebral arteriovenous malformations (CAVMs) are rare vascular anomalies that can significantly impact neonatal morbidity and mortality. These malformations are characterized by an abnormal direct connection between cerebral arteries and veins, bypassing the capillary network. This arteriovenous shunting leads to disorganized vascular architecture and abnormally low resistance to blood flow, which may compromise cerebral perfusion and cardiac function in the neonate, leading to high-output cardiac failure and significant neonatal morbidity and mortality [29].

CAVMs can be classified according to their location, their structure, and the gestational age at which they present [30].

The location can be in the pial, dural, or choroidal spaces, and this is a key factor in the risk of brain damage. Pial anomalies are mainly supplied by the cerebral arteries, dural anomalies by the dural arteries, and vein of Galen anomalies that develop in the choroidal space are primarily supplied by the pericallosal and choroidal arteries. When drainage occurs into the subpial veins, these veins connect with the subependymal veins, and if blood pressure increases, there may be a higher risk of brain tissue damage. In CAVMs that drain mainly into the subarachnoid veins, venous flow can quickly drain into the dural sinuses, resulting in a lower risk of brain damage [29,30].

Regarding structure, when there is a complex vascular structure between the arterial and venous systems, it is called a “nidus” type anomaly. When there is no such structure and direct communication exists between an artery and a vein, it is called a “fistula” type anomaly [30].

Regarding the timing of diagnosis, as already mentioned, diagnosis during fetal life is rare. The most common anomaly diagnosed prenatally is Vein of Galen aneurysmal malformation (VGAM), which typically appears as a single malformation and occurs in fewer than 1 in 25,000 births [30]. Rarely, CAVMs may manifest as multiple vascular malformations, and the risk of cardiac failure is higher due to an increased cardiac output burden [29].

Genetic alterations are now recognized as contributing factors in the pathogenesis of CAVM, with several implicated genes identified. These genes are involved in the regulation of angiogenesis, and mutations occurring during early stages of organogenesis can disrupt normal vascular development. The earlier the mutational event, the higher the likelihood of multiple malformations, often as part of systemic vascular syndromes such as hereditary hemorrhagic telangiectasia or capillary malformation–arteriovenous malformation syndrome [29].

This text will focus on VGAM, which, although rare, is the most frequently diagnosed cerebral vascular malformation in fetal life.

### 6.2. Role of Ultrasound and Doppler in Diagnosing VGAM

VGAM results from dilation of the median prosencephalic vein of Markowsky, which is the embryonic precursor of the vein of Galen. Around 7–8 weeks of gestational age, the multiple arteries supplying the tectal plate are located within the primitive meninges and are connected by a vast capillary network that involutes as the subarachnoid space develops at the end of the first trimester. When VGAM occurs, the vascular pattern observed resembles this, including venous return, which reflects an early developmental stage [30]. In nearly all cases of fetal VGAM diagnosis, this anomaly is classified as a nidus anomaly, characterized by a disorganized vascular structure between the arteries and the true aneurysmal sac, with multiple arteriovenous shunts and venous drainage mainly through the straight sinus [30]. In a small proportion of VGAM cases, an association with genetic mutations has been identified. The genes involved include RASA1 and EPHB4, which have also been found in cases of recurrent malformation [30].

**Figure 5:**
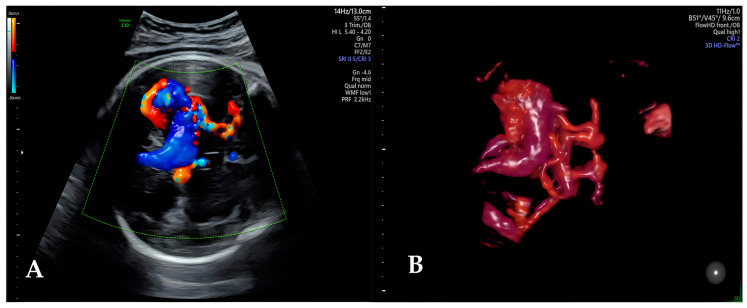
Vein of Galen aneurysmal malformation. (**A**)—Adding color Doppler to B-mode, a vascular structure with prominent turbulent flow is observed in the posterior region of the fetal brain. (**B**)—3D reconstruction of the vascular anatomy, showing abnormal arterial feeding vessels supplying the large vein of Galen.

Before the year 2000, VGAM had a neonatal mortality rate of over 90%, and before the advent of ultrasonography, the diagnosis was made through postmortem anatomopathological examination in approximately 85% of cases [31]. Currently, most VGAM diagnoses are made in the third trimester, although it can be diagnosed in the second trimester. Ultrasound enables suspicion of this diagnosis by observing, in the axial plane, a midline tubular hypoechoic image in the posterior part of the third ventricle, described as a “comet tail” or “keyhole sign.” The differential diagnosis of such an image includes cystic lesions, like arachnoid cysts or porencephalic cysts, choroidal papillomas, and tumors. The use of color Doppler helps to differentiate these conditions and establish the diagnosis of VGAM as it highlights a high-flow vascular structure [30,31,32,33] (Figure 5). However, the ideal way to characterize this anomaly is through transvaginal ultrasound using 3D reconstruction of vascularization with selective display of color/power Doppler signals [30]. This technique allows characterization of the architecture (“nidus” or “fistula” type), the arterial vessels that supply the anomaly, and the venous drainage. Typically, the VGAM is vascularized by arterial branches of the ACA, the pericallosal arteries, PCA, and the choroidal arteries [30,32]. Venous drainage is variable and may occur through the straight sinus, which can be as dilated as the aneurysm itself, or through the persistent falcine sinus. The dural sinuses may also be dilated depending on the intensity of the venous flow draining the lesion [30].

The diagnosis of VGAM necessitates differential diagnosis with other vascular anomalies, namely with the pial variant of arteriovenous cerebral malformation. Doppler marking helps to differentiate these two conditions, although an accurate diagnosis may be difficult in fetal life. A location other than the midline, as well as drainage through the subdural sinuses and not through the straight sinus, are Doppler characteristics that favor another arteriovenous malformation other than VGAM [28].

### 6.3. Hemodynamic Assessment of VGAM Using Doppler

Importantly, because this factor is linked to high morbidity and mortality, ultrasound and Doppler technology enable us to assess how the lesion impacts fetal hemodynamics. As already mentioned, since this anomaly results in a circulatory state of high cardiac output, it is vital to determine the extent of cardiac decompensation. Echocardiographic findings may include cardiomegaly, tricuspid regurgitation, reverse blood flow in the aortic isthmus, and in advanced stages of heart failure—conditions associated with high mortality risk—ascites, pleural effusion, skin edema, and polyhydramnios [30]. Rarely, VGAM may be associated with cardiac malformations such as atrial septal defect, sinus venosus type, and partial abnormal pulmonary venous return. Therefore, echocardiography should also be used to exclude these anomalies.

### 6.4. Ultrasound and Doppler Parameters Associated with Prognosis

Ultrasound and Doppler technology enable the diagnosis and characterization of VGAM, as well as the establishment of a prognosis. The two ultrasound factors that indicate a worse prognosis are ischemic lesions and heart failure.

White matter changes, observed in nearly one-third of cases, are especially evident in second-trimester diagnoses. These changes can manifest as focal hypoechoic lesions, periventricular leukomalacia, or progressive cerebral atrophy, which result from ischemia caused by blood flow diversion due to the VGAM or from compressive effects. Ventriculomegaly occurs in up to 50% of cases. It may be caused by obstruction from compression of the VGAM, high venous pressure disrupting cerebrospinal fluid reabsorption, or secondary to intraventricular hemorrhage, potentially worsening white matter damage [29,30,32,33].

If neither brain damage or heart failure are present, other ultrasound Doppler parameters with a worse prognosis have been described: aneurysm sac volume ≥ 20,000 mm^3^, tricuspid regurgitation with flow velocity ≥ 2 m/s, or the mediolateral diameter and area of the major draining vessel measured at the point of its shortest height [30,33].

### 6.5. Doppler Guidance in Fetal Therapy and Perinatal Management

Finally, ultrasound and Doppler are crucial in fetal therapy. Two successful cases have been documented where fetuses were treated with ultrasound-guided embolization of this type of vascular malformation. Candidates for this therapy are those whose ultrasound evaluation indicates a high risk of cardiac decompensation in the first days of life—making postnatal embolization difficult to schedule around the ideal time, which is about five months—yet who do not have established white matter damage. These candidates must also have a gestational age greater than 23 weeks and a fetal MRI showing a medial-lateral width of the draining venous sinus of the malformation (falcine or straight sinus) measuring ≥8 mm at the point of greatest constriction [30,33]. Therefore, Doppler ultrasound is essential for candidate selection and for fetal therapy.

## 7. Screening of Fetal Brain Malformations

Congenital brain malformations, affecting approximately 1 in 1000 births in Europe, are often associated with severe neurological impairment and significant morbidity. While most cases are diagnosed in the second trimester, a few conditions, such as alobar holoprosencephaly, the acrania–exencephaly–anencephaly sequence, and cephaloceles, can be identified as early as the first trimester [34].

Other central nervous system anomalies may be suspected in the first trimester when specific intracranial structures are systematically evaluated by experienced operators. These include severe ventriculomegaly, corpus callosum anomalies, posterior fossa malformations, and Chiari II malformation. Doppler imaging adds diagnostic value, as from 11 weeks onwards it is possible to visualize the carotid arteries, ACA and its branches in the sagittal plane, as well as the entire Circle of Willis, including the ACA, MCA, and PCA, in the axial plane [34]. In the sagittal plane, several intracranial venous structures can also be visualized in the first trimester. These include the superior sagittal sinus beneath the calvarium, the vein of Galen anteriorly, continuing into the straight sinus at the level of the cerebellar tentorium, and the torcular Herophili posteriorly [34].

For example, Volpe et al. demonstrated that in the sagittal plane of the fetal head in the first trimester, when the choroid plexus is not visible and a cystic alteration of the posterior fossa is suspected, a small angle between the brainstem and the straight sinus (which marks the position of the tentorium) may be an indicator of a Dandy-Walker malformation [34].

**Figure 6:**
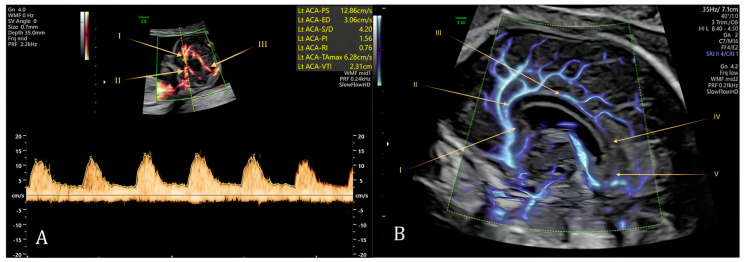
(**A**)—Assessment of the path and flow wave of the anterior cerebral artery/pericallosal artery (I) in the first trimester of pregnancy using Power Doppler. The corpus callosum is not yet visible. Other vascular structures can be observed, such as the internal carotid artery (II) and the straight sinus (III). (**B**)—Later in pregnancy, the anterior cerebral/pericallosal artery is observed running along the entire length of the corpus callosum (I—rostrum, II—genu, III—body, IV—isthmus, V—splenium).

Conturso et al. demonstrated that the pericallosal arteries can be consistently visualized in normal fetuses from 11 weeks of gestation. In most cases, their full course, following the contour of the developing corpus callosum, can be observed (Figure 6). Early visualization of these arteries appears to correlate with normal corpus callosum development, which only becomes sonographically apparent from 18 weeks onward. Conversely, non-visualization of the pericallosal arteries in the first trimester may serve as an early marker of agenesis. In cases of corpus callosum dysgenesis, the arteries are often present but display abnormal morphology and trajectory [35]. Other groups have published data consistent with these findings, concluding that visualization of the pericallosal arteries is an indirect but reliable sign to exclude corpus callosum agenesis on first-trimester ultrasound [34,35,36].

In the assessment of corpus callosum anomalies, a quantitative Doppler-based marker, the TACAD (Tela-Choroidea-to-Anterior-Cerebral-Artery Distance), has been proposed by Karl and Chaoui. This parameter measures the distance between the inferior margin of the tela choroidea and the A3 segment of the ACA as it curves around the genu of the corpus callosum. Evidence suggests that TACAD values decrease proportionally with the severity of callosal anomalies. The same authors have published reference curves for TACAD in normal fetuses during the second half of gestation [37].

In the second trimester, an abnormal ACA trajectory has also been identified in some works as a marker of other fetal brain anomalies that are sometimes linked to difficult ultrasound diagnosis.

Bernard et al. published a clinical case describing a fetus at 24 weeks of gestation with a cavum septum pellucidum anomaly and an inability to visualize the corpus callosum, but with apparently normal division of the cerebral hemispheres. Color Doppler demonstrated an abnormal course of the ACA along the inner side of the frontal bone, a sign described as “snake crawling under the skull,” which was later diagnosed as lobar holoprosencephaly by magnetic resonance imaging and confirmed by postmortem examination [38]. Shortly thereafter, other cases were described by Blin et al. demonstrating this same sign and its relationship to the diagnosis of lobar holoprosencephaly [39]. More recently, Delmas et al. published another case of absence of the cavum septum pellucidum and altered morphology of the corpus callosum associated with a vertically oriented ACA. The MRI study showed, in addition to the corpus callosum alterations, fusion of the posterior region of the frontal lobes and the anterior region of the parietal lobes, features consistent with a diagnosis of syntelencephaly [40]. Although a detailed description of pericallosal artery anatomical variants falls outside the scope of this text, it is important to note that five distinct patterns have been described in fetal life. Familiarity with these variants is essential for the accurate interpretation of deviations suggestive of pathology [41].

Finally, in cases of congenital cytomegalovirus infection and resulting structural anomalies, ultrasound can detect a vascular change called striatal artery vasculopathy. This condition involves hyalinization or mineralization of the striatal arteries, which are branches of the MCA that supply the germinal matrix. Normally, these vessels are not visible; however, when vasculopathy occurs, one or more linear echogenicities may be seen [5].

## 8. Fetal Growth Restriction

Fetal growth restriction (FGR) is likely the clinical condition where Doppler technology is most commonly applied in obstetrics, from definition and risk assessment to screening, diagnosis, and management.

The term FGR applies to all fetuses that do not reach their full growth potential and is estimated to occur in approximately 3–9% of pregnancies in the developed world and up to 25% of pregnancies in the non-developed world [42,43]. However, with the tools available in the clinic, accurately assessing a fetus’s true growth potential remains challenging. The older criterion for defining FGR—estimated fetal weight (EFW) or abdominal circumference (AC) below the 10th percentile for gestational age—has been supplemented by criteria based on Doppler flowmetry of fetal vascular territories. These additional criteria help differentiate fetuses that are constitutionally small for gestational age from those with actual growth restriction, who face a higher risk of adverse outcomes. Therefore, most societies, but not all, currently adopt the criteria shown in Table 1 as the definition of FGR [42,43,44,45]. With these criteria in mind, there are some important aspects to consider. First, in early FGR, diagnosed before 32 weeks, placental dysfunction typically results from impaired trophoblastic invasion, leading to abnormalities mainly seen in uterine artery (UtA) and umbilical artery (UA) flowmetry. In late FGR, diagnosed after 32 weeks, the placenta cannot fully meet the fetal metabolic needs, causing fetal redistribution of blood flow, especially toward the fetal brain. The main Doppler abnormalities are a decreased cerebroplacental ratio (CPR) and, less often, an increased UA PI. Second, although the risk of poor outcomes increases for all fetuses with EFW below the 10th percentile, the highest risk for morbidity and mortality, especially neurodevelopmental impairment, is found in fetuses below the 3rd percentile. Evidence also indicates that, aside from the estimated weight percentile, growth velocity is an important factor [42,45]. Several studies have shown that lower growth velocity correlates with increased adverse outcomes in both small-for-gestational-age fetuses and those with EFW above the 10th percentile [44].

Villalaín et al. suggest two additional criteria for diagnosing late FGR beyond EFW below the 10th percentile: soluble Fms-like tyrosine kinase-1 (sFlt-1)/placental growth factor (PlGF) ratio greater than 38, since this ratio indicates an imbalance between anti-angiogenic and pro-angiogenic factors, with a dominance of the former; and UA PI above the 95th percentile, as evidence shows this is linked to a higher risk of adverse outcomes [44].

The importance of risk assessment, screening, diagnosis, and monitoring of fetuses with growth restriction relates to the increased risk of neonatal morbidity and mortality. FGR is linked to a higher risk of stillbirth, iatrogenic prematurity, neonatal death, perinatal asphyxia, respiratory distress syndrome, hypothermia, hypoglycemia, polycythemia, jaundice, pulmonary hemorrhage, late-onset sepsis, feed intolerance, necrotizing enterocolitis, growth retardation, behavioral disorders, and significant or subtle neurodevelopmental impairments with cognitive and learning disabilities. In addition to this rise in childhood morbidity, FGR is also associated with developing pathology in adulthood due to fetal epigenetic phenomena, such as metabolic syndrome and cardiovascular conditions [40]. Once diagnosed, FGR has no direct treatment, but preventive strategies are available, and proper monitoring along with determining the location and timing of delivery have consistently been shown to improve the prognosis of newborns, reducing the stillbirth rate by about 50% [44].

There are several causes of FGR, such as chromosomal abnormalities, genetic syndromes, and infections, but the most common cause is placental dysfunction. We will focus on this last context, as this is where the Doppler study becomes particularly relevant.

Regarding screening, this can happen in three stages: during the first trimester to identify women who might benefit from preventive measures, during the second trimester to detect early FGR cases, and during the third trimester to identify late FGR cases.

Regarding first-trimester screening, evidence shows that combining maternal history, mean arterial pressure (MAP), Pregnancy-Associated Plasma Protein-A (PAPP-A), Placental Growth Factor (PlGF) values, and UtA PI detects 34.3%, 48.6%, and 59.1% of cases of birthweight below the 10th percentile at term, before 37 weeks, and before 32 weeks’ gestation, at a 10% false positive rate. The respective detection rates for birthweight below the 3rd percentile are 39.9%, 53.2%, and 64.4%, and for birthweight below the 3rd percentile with preeclampsia, 46.3%, 66.8%, and 80.4%. In general, universal screening is not yet recommended. However, it is advised that women at high risk of developing early preeclampsia have an ultrasound plan to monitor fetal growth, as this group accounts for half of newborns weighing less than the 10th percentile. Additionally, in this group, starting low-dose aspirin up to 16 weeks of gestation reduces both the incidence of preeclampsia and rates of FGR and perinatal death [44,45,46,47].

**Figure 7:**
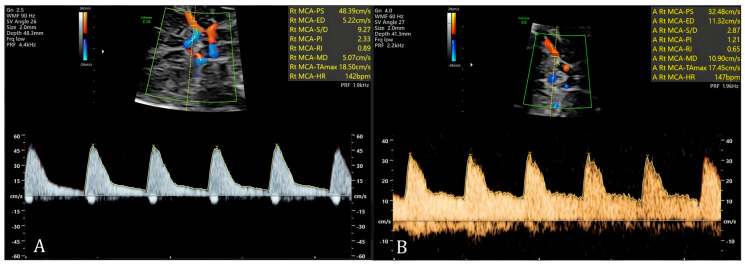
Flow in the middle cerebral artery in the third trimester in a fetus without growth restriction (**A**) and in a fetus with growth restriction (**B**). Note the decrease in the pulsatility index (MCA-PI) in the fetus with growth restriction, reflecting the increase in diastolic flow visible in the graphical representation of the flow wave—“brain sparing” effect.

Regarding second-trimester FGR screening, Doppler technology becomes relevant again. Compared with other methods, measurement of the UtA PI shows a 60% early FGR detection rate for values above p90 (Figure 7). If combined with reassessment of EFW at 26 weeks, it also performs well in detecting both early preeclampsia and FGR. Therefore, the UtA PI measure should be considered a screening method for early FGR, leading to a growth surveillance plan for high-risk patients starting around 26 weeks [42,47,48]. As in the second trimester, combining Doppler with EFW, which ideally should be performed at 35–37 weeks of gestation, increases the screening capacity for late FGR [44].

Once the diagnosis of FGR is made, there is no consensus among different societies on the follow-up plan. Still, generally, it is recommended to assess fetal growth every 2 to 4 weeks, along with the assessment of flow in the UA, since this assessment has been shown to decrease the risk of perinatal death, labor inductions, and cesarean sections. Assessment of the cerebroplacental ratio (CPR), which evaluates the fetal “brain-sparing” effect, should also be considered since it is strongly associated with increased perinatal morbidity and mortality [42,45]. The frequency of Doppler re-evaluations should then be based on the severity of FGR and changes in flow in the UA [42]. As UA IP increases, diastolic flow decreases, and in extreme cases, it may become absent or even reversed. Subsequently, changes in ductus venosus flow parameters may occur, reflecting the end of the sequence of hypoxemia, hypoxia, and acidosis. These changes, detectable by Doppler, allow monitoring the deterioration of the fetal metabolic and hemodynamic status and determining the optimal time and mode of delivery, based also on gestational age [42,49].

Twin pregnancies are considered complicated by FGR when one fetus has an EFW below p10 and the EFW discrepancy between the two is 25% or greater. In bichorionic pregnancies, ultrasound monitoring is identical to that of a singleton pregnancy, but in monochorionic pregnancies, Doppler imaging once again plays a central role [50]. According to Gratacós, selective FGR in monochorionic pregnancies can be divided into three types according to the UA flow pattern, and these three patterns present significant differences with regard to evolution and perinatal outcome [51]. In Type I, the UA Doppler waveform has positive end diastolic flow (EDF). In Type II, there is absent or reversed end-diastolic flow (AREDF). In Type III, there is a cyclical pattern of positive EDF and AREDF [50]. Although there is no solid evidence on the best way to manage these cases (conservative management followed by early delivery, laser ablation, or selective termination of the growth-restricted twin with the aim of improving the perinatal and long-term outcome of the other twin), surveillance involves serial assessment of fetal growth and flowmetric evaluation, particularly of the UA, with the aim of determining the ideal gestational age for termination of pregnancy and, above all, avoiding intrauterine death of the fetus with growth restriction [50,51,52].

## 9. Fetal Anemia

The use of the middle cerebral artery peak systolic velocity (MCA-PSV) for diagnosing and managing fetal anemia was one of the discoveries that significantly transformed clinical practice in obstetrics. The first studies in this field date back to 1987, and all research since then has resulted in about a 70% reduction in invasive tests conducted to diagnose fetal anemia [53]. Before its use, diagnosing and managing fetal anemia relied on the technique described by Liley in 1961, which measured bilirubin in amniotic fluid with a spectrophotometer [53]. The measurement of maximum systolic velocity in the MCA is based on the fact that, in anemic fetuses, blood flow velocity is higher than in non-anemic fetuses because of lower blood viscosity and the hyperdynamic circulation that develops to meet the fetus’s metabolic needs. By prioritizing vital organs, such as the brain, the MCA serves as an excellent indicator of fetal adaptation to anemia.

Fetal anemia is diagnosed when the fetal hemoglobin level is two standard deviations below the mean for gestational age or when the hematocrit is below 30%. The reference values for fetal life are described and published for gestational ages between 18 and 40 weeks [54].

The primary cause of fetal anemia is maternal alloimmunization. In this condition, maternal sensitization to alloantigens occurs, leading to the production of antibodies that can react against the surface antigens of fetal red blood cells during pregnancy. This reaction results in their destruction and subsequent anemia. Non-immune causes of fetal anemia include parvovirus infection, hereditary anemias, malignancies, and hemorrhage [54]. Another cause is twin anemia–polycythemia sequence (TAPS), which results from intertwin blood transfusion through very small placental anastomoses that happen chronically and insidiously, creating a significant hemoglobin difference between twins without the classic, more noticeable signs of twin-to-twin transfusion syndrome [55].

The importance of screening and diagnosing anemia in high-risk fetuses is because severe anemia is linked to the development of high-output heart failure, hydrops, and death. Timely diagnosis allows for proper monitoring and treatment, which currently includes immunomodulation, intrauterine transfusion, or planned delivery.

**Figure 8:**
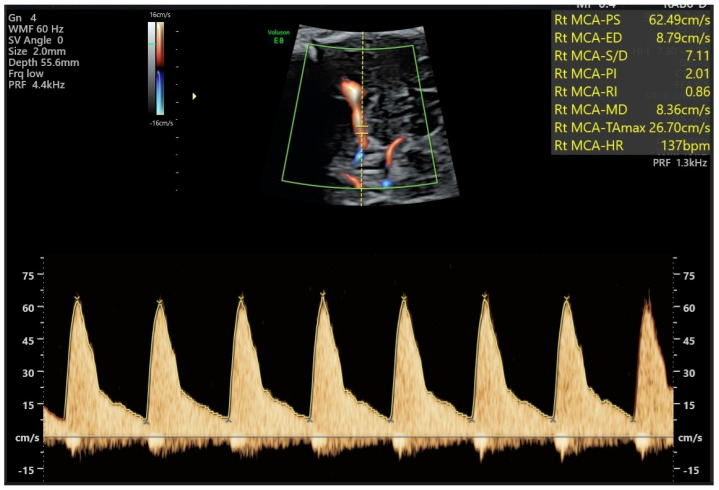
Doppler evaluation of the flow wave in the middle cerebral artery. The Circle of Willis is identified using color Doppler; the color box is positioned over the artery and zoomed in. The gate is placed over the middle cerebral artery, centered within the vessel just after its origin from the internal carotid artery, at an angle close to zero, with the fetus at rest. The flow waveforms obtained are consistent, and the point of maximum wave velocity is recorded (MCA-PS).

The sensitivity of MCA PSV for diagnosing fetal anemia in high-risk fetuses varies from 7% to 100%, depending on the operator’s experience, making proper use of this tool crucial. An axial plane of the fetal head should be obtained, including the thalami, cavum septi pellucidi, and greater wing of the sphenoid. The Circle of Willis should be identified with color Doppler, and the color box should be positioned over the MCA and zoomed in. The sample should be placed over the MCA, centered within the vessel just after its origin from the internal carotid artery, at an angle close to zero, as this reduces intra- and interobserver variability. The fetus should be at rest, since heart rate accelerations and movements can affect measurements. The flow waveforms obtained should be consistent, and the point of maximum wave velocity should be recorded. This procedure should be repeated three times (Figure 8) [53,56].

MCA-PSV is affected by various factors such as fetal activity, heart rate, gender, myometrial activity, advanced gestational age, history of previous transfusions, altered placentation, FGR, and, as already mentioned, inadequate technique. These factors explain the presence of false positives and false negatives in fetal anemia screening; therefore, the results should be interpreted while considering all these elements [56].

Evidence indicates that an MCA-PSV exceeding 1.50 MoM in fetuses at high risk of anemia, without hydrops, identifies nearly all cases of moderate to severe anemia, with a false-positive rate of 12% [56]. This effectiveness has been proven not only in alloimmune anemia but also in infectious anemia, twin-twin transfusion syndrome, fetal hydrops, and fetal-maternal hemorrhage [53,56,57]. Additionally, this method has shown to be superior to the previous technique of measuring bilirubin levels in amniotic fluid [58]. An added advantage of MCA-PSV evaluation over amniotic fluid spectrometry is its capacity to diagnose anemia in cases of anti-Kell isoimmunization, where the primary cause is the suppression of red blood cell precursors in the bone marrow rather than hemolysis. Mari et al. also demonstrated the effectiveness of MCA Doppler in this scenario [53].

Doppler technology can also help reduce false negatives in MCA-PSV assessment. In severe anemia, tricuspid regurgitation often precedes ascites and hydrops. Therefore, if an at-risk fetus has a normal MCA assessment but a cardiac Doppler showing tricuspid regurgitation, a surveillance plan should be established to monitor for anemia [53,59].

Once an MCA-VPS greater than 1.5 MoM is detected, ultrasound and Doppler serve as tools that enable a definitive diagnosis of fetal anemia, guiding umbilical cord puncture for fetal blood sampling. The criteria for diagnosing moderate to severe anemia may differ between Fetal Medicine Centers, but generally, it is diagnosed when the hemoglobin concentration is 4 to 5 standard deviations below the mean for gestational age, a hemoglobin deficit greater than 5 g/dL, an absolute hemoglobin concentration less than 10 g/dL, or a hematocrit less than 30%.

Intrauterine transfusion (IUT) is also performed under continuous ultrasound control, ideally by puncturing the umbilical vein at the site of cord insertion in the fetal abdomen, in its intrahepatic portion. The transfused blood consists of fresh donor red blood cells, 0-negative, CMV-negative, washed, irradiated, leukocyte-depleted, with hematocrit between 75 and 80%, negative for possibly immunizing antigens, with the aim of increasing fetal hematocrit by up to 40–50% and taking into account a fetal blood count previously performed in the same procedure [58].

Although the MCA-PSV assessment is also used as monitoring after IUT, its sensitivity for predicting moderate to severe anemia decreases with the number of IUTs performed, being estimated at 78% (63–88%), 74% (48–90%) and 60% (34–82%) for one, two and three transfusions [59].

In conclusion, fetal anemia, mostly of hemolytic origin, continues to be an obstetric challenge, significantly contributing to obstetric, fetal, and neonatal morbidity, as well as health care costs. However, thanks to changes in screening, diagnosis, and treatment practices—with Doppler technology playing a key role—survival rates are now as high as 95%, a notable improvement over previous rates [56,60].

## 10. Anastomoses in Monochorionic Twins and Twin-Twin Transfusion Syndrome

Monochorionic pregnancies involve twins who share a single placenta. They occur in approximately 0.4% of all pregnancies and represent about 10–15% of all twin pregnancies. These cases warrant special attention because they are frequently associated with complications that may lead to significant morbidity and mortality [61,62,63].

Some complications are unique to monochorionic pregnancies because of vascular anastomoses connecting the placental territories of each fetus. These anastomoses may be arterio-arterial (AA), veno-venous (VV), or arteriovenous (AV). The AA and VV anastomoses are superficial, allowing bidirectional blood flow between the twins, whereas AV anastomoses are deep and permit unidirectional flow. When there is hemodynamic equilibrium between the two fetal circulations, the pregnancy often progresses without major complications. However, if this balance is disrupted and excess blood flow occurs toward one fetus through AV anastomoses, twin–twin transfusion syndrome (TTTS) may develop. In some cases, this imbalance can be partially compensated by flow through AA anastomoses. A more subtle form of hemodynamic imbalance occurs in twin anemia–polycythemia sequence (TAPS), which results from tiny AV anastomoses, typically less than one millimeter in diameter [58]. Timely diagnosis of TTTS, facilitated by proper ultrasound monitoring, enables effective treatment through fetoscopic laser surgery. This approach results in the survival of at least one fetus in 70% of cases but increases the risk of preterm birth [63].

To interpret the ultrasound findings of twin–twin transfusion syndrome (TTTS), it is essential to understand the underlying pathophysiology. The recipient twin, who receives an volume of blood, experiences increased preload. The resulting stretching of the cardiac chambers stimulates the release of atrial natriuretic peptide (ANP) and brain natriuretic peptide (BNP), which enhance urine output and consequently increase amniotic fluid volume. Simultaneously, endothelin levels rise, contributing to hypertension, cardiac hypertrophy, and valvular regurgitation. In contrast, the donor twin, affected by hypovolemia, activates the renin–angiotensin–aldosterone system (RAAS), leading to reduced urine output and oligohydramnios. Because vasoactive substances such as renin and angiotensin II can cross the placental vascular anastomoses, they may further exacerbate hypertension and cardiac dysfunction in the recipient twin [63,64].

Ultrasound may reveal several findings such as differences in bladder filling between fetuses, variations in amniotic fluid levels between amniotic sacs, Doppler anomalies of the umbilical artery, ductus venosus and umbilical vein, hydrops and even death.

Recipient fetuses more often show signs of right ventricular hypertrophy, impaired diastolic function, and tricuspid regurgitation, which can then lead to functional subvalvular right ventricular outflow obstruction. These changes are reflected by Doppler shifts in venous flows. Donor fetuses more often show signs of increased placental resistance, which result in Doppler changes in the umbilical artery. They less frequently exhibit structural cardiac changes, but prolonged hypovolemia can lead to coarctation of the aorta due to reduced flow in the aortic isthmus [63]. Thus, ultrasound and Doppler technology allow the diagnosis of TTTS and its staging.

Quintero described a Staging System in 1999, which, due to its reproducibility, remains universally recognized as a method for monitoring these fetuses. Fetal echocardiography can be useful in recognizing the cardiac alterations that lead to the abnormalities described in the various stages and in pre- and postoperative evaluation. Regarding the Quintero System, Stage I was defined as visualization of the bladder of the donor twin and amniotic fluid discrepancy between the two fetuses (maximum vertical pocket of amniotic fluid greater than 8 cm and oligohydramnios for the donor twin with a maximum vertical pocket less than 2 cm); stage II involves the failure to visualize the bladder of the donor fetus for at least 60 min; in stage III, significant Doppler alterations are observed: AREDF in the UA or ductus venosus or pulsatile umbilical venous flow; stage IV corresponds to hydrops; and stage V, fetal death of one or both fetuses [63,65,66,67,68,69,70,71,72].

Doppler is also essential in surgical mapping prior to laser ablation of placental anastomoses; after determining the placental borders, Doppler allows determining the insertion site of the umbilical cords, as well as excluding velamentous insertion of the cords, either to determine the best location to construct the placental neoequator (which should ideally be equidistant from the cord insertions), or to decrease the likelihood of vascular damage [63,66].

After treatment, ultrasound and Doppler also allow for necessary monitoring; they allow for monitoring resolution, and also allow for the detection of recurrences or the onset of new pathological conditions such as TAPS, which can develop due to the maintenance of very small anastomoses [66,67].

As mentioned previously, anastomoses smaller than 1 mm can lead to transfusion between fetuses without discrepancies in amniotic fluid volumes. This situation is detected by the MCA Doppler study, which allows anemia in the donor fetus to be diagnosed. The MCA Doppler is not currently validated for the diagnosis of polycythemia in the recipient fetus. In addition to an MCA-PSV value above 1.5 MoM being a predictor of anemia, the difference in MCA-PSV between the two fetuses has been documented as the best predictor of a significant difference in hemoglobin values between newborns. Therefore, a diagnosis of TAPS is accepted if there is an absolute MCA PSV above 1.5 MoM for the donor and below 1 MoM for the recipient, or if there is a between both of 1 MoM [59]. The staging of TAPS is also based on the Doppler study: stage 1 exists when MCA-PSV ≥ 1.5 MoM in the donor and ≤1.0 MoM in the recipient (or difference between both in MCA-PSV higher than 0.5 MoM), stage 2 when MCA-PSV ≥ 1.7 MoM in the donor and ≤0.8 MoM in the recipient (or difference between both in MCA-PSV higher than 0.7 MoM), stage 3 when there are Doppler alterations in the fetal territories (AU and ductus venosus), stage 4 when there is hydrops and stage 5 when there is fetal death [66].

TAPS can be treated with laser surgery, but management options include expectant management with ultrasound surveillance and fetal blood transfusion of the donor, with or without partial exchange transfusion of the recipient fetus. In all of these options, the role of a Doppler is unquestionable [66,73].

## 11. Abnormal Neurodevelopment in Childhood

The formation of the human brain, encompassing all the processes that transform the microscopic neural tube into a highly complex organ composed of billions of specialized cells interconnected within a neural network governed by intricate molecular interactions, remains a rapidly evolving field of research. Although many of these processes and interactions are still not fully understood, it is well established that brain development begins during the embryonic period and continues after birth, throughout childhood and adolescence. Its final structure and function depend on a wide range of genetic and environmental factors [74].

Multiple authors have studied the fetal brain to describe normal ultrasound patterns, identify anomalies during the fetal period, and explore their relationship with psychomotor development. Normal brain development follows a predictable sequence throughout the embryonic and fetal stages.

Ultrasound evaluation throughout pregnancy allows observation and documentation of the sulcation process, which is evident from the end of the first trimester. Several authors have developed descriptive and quantitative methods to describe the development of the cerebral convolutions, including scoring systems to make this assessment feasible and reproducible [75,76,77,78]. However, a delay exists between actual anatomical development and its visualization on imaging. This delay may range from two to six weeks, as observed with the parieto-occipital fissure and the calcarine sulcus, which become visible on imaging approximately two weeks after their anatomical formation [75].

Deviations from normal patterns indicate fetuses at risk for neurodevelopmental anomalies. One example is the altered angles of the Sylvian fissure, described by Poon and colleagues, which may occur before the diagnosis of cortical developmental abnormalities, thereby signaling fetuses at risk [76,79].

One of the most studied causes of fetal brain development abnormalities is FGR. After neurogenesis is complete, axonal growth, dendritic branching, and the development of synaptic activity continue. These processes are compromised in fetuses with FGR, leading to decreased cortical volume, altered cortical organization, and reduced connectivity between circuits, which results in suboptimal cognitive development after birth [80]. Multiple efforts have been made to develop tools that allow establishing the prognosis in these situations.

As previously described, on one hand, the development of Doppler technology allows mapping of cerebral vessels during practically the entire gestation; on the other hand, Doppler alterations of fetal vessels in FGR situations are well described; thus, in addition to the important role of Doppler in optimizing perinatal outcomes, Doppler emerges as a potential tool for predicting neurological development [81,82,83,84].

Delorme and colleagues studied a population of pregnant women with hypertensive disorders, whose fetuses experienced early FGR and required delivery before 32 weeks. Prenatal ARED in the UA was linked to a higher incidence of severe or moderate neuromotor and/or sensory impairment compared to normal or reduced end-diastolic flow in the UA [85].

According to Morsing, in cases of early FGR, survival at 2 years was higher for fetuses with zero diastolic flow in the UA compared to those with reversed flow in the UA. However, regarding neurological development, there was no significant difference in neurodevelopmental impairment rates between the two groups. Gestational age remained the primary factor influencing psychomotor development [86].

Brodszki et al. have shown that for absent or reversed flow in the umbilical artery, if delivery happens before significant changes in ductus venosus flow, there can be high survival rates with low neurologic morbidity [87].

According to the GRIT study, neurological outcomes at two years of age appear to be better in fetuses younger than 30 weeks when delivery is delayed until the obstetrician considers it necessary. This improvement may be related to changes in the umbilical artery, although the study did not perform a subanalysis of the different flow wave patterns in the umbilical artery [88].

The TRUFFLE Study was the first prospective, randomized study to examine neurological outcomes according to the decision to deliver fetuses with early FGR based on Doppler changes (changes in short-term variation on cardiotocography, pulsatility index above the 95th percentile in the ductus venosus, A wave at or below baseline in the ductus venosus). This study showed no significant differences in neurological outcomes between the three groups; however, there was a trend toward decreased neurological impairment in the third group. The authors conclude that deferring delivery until late changes in the ductus venosus occur (unless delivery is mandated earlier by the CTG safety criteria) compared with delivery based solely on short-term CTG variation possibly results in a small excess of antenatal deaths but also in substantially improved survival without impairment at 2 years of age, corrected for prematurity [89].

Ganzevoort and colleagues compared data from these two studies and verified the hypothesis that in order to optimize neurological outcome at two years of age in fetuses with early FGR, the optimal form of monitoring includes computerized analysis of cardiotocography and Doppler of the ductus venous [90].

Mazarico and colleagues developed a predictive model to enhance parental counseling for fetuses with early FGR, focusing on mortality and severe neurological morbidity. This model included gestational age at delivery, male sex, and Doppler stage within 48 h before birth: (1) positive UA- end diastolic flow (EDF) and DV PI ≤ 95th centile; (2) absent UA EDF; (3) reversed UA EDF or DV PI > 95th centile; and (4) absent or reversed DV EDF. For predicting mortality or severe neurological morbidity, the model achieved a sensitivity of 55%, negative predictive value of 63%, and positive predictive value of 74%, at a 20% false-positive rate. This performance markedly outperforms counseling based solely on gestational age [91].

Regarding late FGR, the pathophysiological mechanism, as previously mentioned, involves placental alterations that do not result in increased placental resistivity, so no changes in UA flowmetric indices are expected. However, these fetuses show neurological vulnerability: they are at a developmental stage in which the brain’s oxygen demands are higher, making them less tolerant to hypoxia. Consequently, in addition to higher morbidity and mortality, they also face an increased risk of suboptimal neurodevelopment.

In terms of Doppler findings, the indices that best characterize these fetuses are the MCA PI and the cerebroplacental ratio (CPR), which reflect cerebral vasodilation. Changes in CPR may precede a decrease in MCA PI, making this ratio a more sensitive tool for earlier detection of cerebral vasodilation. Even with normal CPR values, a downward trend could indicate the beginning of fetal hypoxia. However, the evidence regarding the relationship between fetal cerebral hemodynamic indices and neurological outcome is still inconsistent.

According to some authors, CPR changes are linked to a higher risk of alterations in neonatal motor activity, lower communication scores, diminished problem-solving abilities at 2 years of age, poorer cognitive development with worse coefficients of global development [86,92]. 

However, Sun et al. found no association between the findings of MCA Doppler assessment, i.e., PI or CPR, and brain structure or neurological outcome in newborns delivered at term. These authors suggest that vasodilation near term represents an effective adaptive response, since this group of newborns presented neurological outcomes similar to the average of newborns with appropriate weight at 18–36 months [93].

## 12. Discussion

This literature review aims to underscore the pivotal role of Doppler ultrasonography in the assessment of fetal cerebral anatomy and in the evaluation of fetal pathologies characterized by hemodynamic alterations reflected in cerebral vascularization.

Recent technological advances, coupled with an expanding understanding of fetal physiology and pathophysiology, have significantly enhanced our ability to identify ultrasound and Doppler abnormalities at increasingly earlier stages of gestation.

One illustrative example is the introduction of new Doppler modalities, such as slowFlow Doppler, which enables visualization of blood vessels based on flow characteristics. This technique allows for differentiating true blood movement from surrounding tissue motion, while maintaining an excellent fetal safety profile [94]. Additionally, the LumiFlow modality supports three-dimensional reconstructions of the fetal cerebral vasculature, achievable as early as the end of the first trimester, providing unprecedented spatial detail in early neurovascular development assessment [81]. These modalities will likely influence the future of fetal neuroimaging and facilitate earlier characterization of cerebral hemodynamics.

However, has the implementation of Doppler ultrasonography in obstetric practice translated into an absolute reduction in fetal mortality? According to studies by Grytten et al. and Brown, the introduction of obstetric ultrasound into routine clinical care has indeed contributed to a decline in fetal mortality rates [95,96]. This improvement primarily resulted from the early identification of high-risk pregnancies, including post-term pregnancies (through more accurate gestational dating), multiple gestations, and pregnancies complicated by placental implantation abnormalities.

Regarding the specific contribution of Doppler technology, as early as 1996, Meyberg et al. [97] had already postulated its potential to reduce fetal mortality by enabling timely identification of fetuses at risk and by mitigating the effects of intrauterine hypoxia and acidosis. More recently, in 2021, Grytten and colleagues analyzed the population-level impact of Doppler implementation on fetal mortality. Their findings indicated that the introduction of Doppler studies in obstetric care was associated with an approximate 30% reduction in fetal mortality, accompanied by a 15% increase in the cesarean section rate. This decline in mortality was particularly evident among preterm fetuses, although a decreasing trend was also observed in term and post-term fetuses. The absence of statistical significance in the latter groups may be attributed both to their inherently lower mortality rates and to the lack of systematic Doppler assessment in late gestation [98]. These results are consistent with those already reported by Imdad and colleagues, who reported a reduction in fetal mortality of approximately 29% [99].

The use of cerebral Doppler significantly contributes to this improvement in obstetric outcomes. It enables early diagnosis, the ability to monitor pathological conditions—reducing the need for invasive techniques in some cases—supports fetal therapy techniques, and ultimately, it allows determining the best timing, best place, and best mode of delivery.

To achieve these goals of reducing fetal mortality and optimizing perinatal and long-term outcomes, it is necessary, as with the implementation of any new technology, to establish training and learning programs, as well as audit processes, practices already adopted in the field of obstetrics by international organizations such as the Fetal Medicine Foundation and the International Society of Ultrasound in Obstetrics and Gynecology [100]. As previously mentioned, the sensitivity of cerebral Doppler velocimetry measurements for certain pathological cases is highly dependent on operator training. Additionally, there are published guidelines on the use of cerebral Doppler velocimetry in obstetrics to ensure this practice is standardized and interobserver variability is minimized [101]. With the implementation of standardized procedures, it will also be easier to more reliably evaluate the benefits of cerebral Doppler technology in both fetal medicine and perinatology, as well as in long-term outcomes.

## 13. Future Research

There is no doubt regarding the extensive and well-documented applications of fetal cerebral Doppler ultrasound in Obstetrics. Nevertheless, its potential for future developments remains considerable.

One area in which further research could enhance the clinical value of cerebral Doppler is the investigation of FGR. As previously discussed and reported by several authors, the expected sequential pattern of hemodynamic deterioration is not consistently observed and exhibits substantial interindividual variability [102,103,104]. The development of novel brain hemodynamic indices may, in the future, contribute to a better understanding of this variability among fetuses who, despite sharing the same pathological condition, demonstrate distinct adaptive responses.

Another area of considerable interest concerns the study of FGR in fetuses that are adequate for gestational age. Although these fetuses have estimated weights between the 10th and 90th percentiles, they may fail to achieve their individual growth potential and can thus belong to a subgroup with unexpectedly adverse outcomes. In this context, the investigation of novel brain flowmetric indices may offer additional insights into subtle alterations in fetal hemodynamics. Furthermore, it is well established that, in response to hypoxia, certain cerebral regions receive preferential blood flow. The exploration of new vascular territories may therefore contribute to a better understanding of the mechanisms underlying this selective perfusion and its possible associations with subsequent neurodevelopmental outcomes [105,106,107,108].

Significant progress is also anticipated in the development of Artificial Intelligence (AI) applications for obstetric ultrasound, particularly in Doppler assessment. This technology has the potential to enhance workflow efficiency, reduce operator fatigue and wrist strain, and minimize inter-observer variability, thereby decreasing operator dependency and improving measurement consistency. Its performance in biometric data acquisition is already well documented, with reported success rates ranging from 87% to 100%. In addition, current models already offer a high capacity for image recognition and interpretation [109,110,111]. The same applies to Doppler technology [110]. According to Aguado and colleagues (2024) reported that their models achieved high accuracy in classifying cerebral Doppler views, distinguishing flow waveforms from different vascular territories, and extracting key measurements from acquired images [112]. Similarly, Iftikhar and collaborators demonstrated that the application of AI to Doppler assessment can also be cost-effective [113]. In relation to the development of predictive models for perinatal mortality, a 2022 systematic review indicated that this field remains in its early stages, although it shows substantial promise for future clinical implementation, as we expect to happen with fetal brain Doppler [114]. An additional advantage of AI lies in its capacity to provide real-time feedback to operators, guiding them in obtaining optimal imaging planes. Consequently, AI may, in the future, become an integral component of training programs for professionals specializing in fetal medicine and fetal brain Doppler. Moreover, simulation-based training supported by AI-guided feedback has already been associated with significant improvements in clinical performance [115,116].

## 14. Conclusions

Advances in technology and the growing understanding of fetal medicine have established Doppler assessment of fetal cerebral vessels as a fundamental tool in the monitoring of fetuses with congenital malformations, FGR, anemia, and complications arising from placental anastomoses in monochorionic pregnancies. When appropriately applied, Doppler ultrasound enables the implementation of targeted monitoring and intervention strategies, thereby reducing fetal morbidity and mortality. Moreover, the correlation between Doppler findings and postnatal psychomotor development provides valuable information for prognosis and parental counseling. This is particularly relevant, as early intervention during childhood has been shown to significantly enhance neurodevelopmental outcomes in children with suboptimal development. Looking ahead, continued progress in these domains is expected to broaden the clinical applications of Doppler ultrasound, further strengthening its role in optimizing fetal outcomes and contributing to improved health throughout childhood and adulthood—the ultimate goal of fetal medicine.

## Figures and Tables

**Table 1:** Delphi consensus-based definitions for early and late fetal growth restriction in absence of congenital anomalies. (AC, fetal abdominal circumference; AEDF, absent end-diastolic flow; CPR, cerebroplacental ratio; EFW, estimated fetal weight; GA, gestational age; PI, pulsatility index; UA, umbilical artery; UtA, uterine artery).

Early FGR	Late FGR
<32 weeks GA	GA ≥ 32 weeks
AC/EFW < 3rd centile or UA-AEDF	AC/EFW < 3rd centile
or	or at least two out of three of the following
1. AC/EFW < 10th centile combined with	1. AC/EFW < 10th centile
2. UtA-PI > 95th centile and/or	2. AC/EFW crossing centiles > 2 quartiles on growth centiles
3. UA-PI > 95th centile	3. CPR < 5th centile or UA-PI > 95th centile

## Data Availability

The original contributions presented in this study are included in the article. Further inquiries can be directed to the corresponding author.

## Abbreviations

The following abbreviations are used in this manuscript:

AA	Arterio-Arterial
AC	Abdominal Circumference
ACA	Anterior Cerebral Artery
AEDF	Absent End Diastolic Flow
AI	Artificial Intelligence
ALARA	As Low As Reasonably Achievable
AREDF	Absent or Reversed End Diastolic Flow
AV	arterio-venous
CAVM	Cerebral ArterioVenous Malformations
CPR	Cerebroplacental Ratio
DV	Ductus Venosus
EDF	End Diastolic Flow
EFW	Estimated Fetal Weight
FGR	Fetal Growth Restriction
IUT	Intra Uterine Transfusion
MAP	Mean Arterial Pressure
MCA	Middle Cerebral Artery
MCA-PSV	Middle Cerebral Artery Peak Systolic Velocity
MI	Mechanical Index
PAPP-A	Pregnancy-Associated Plasma Protein-A
PCA	Posterior Cerebral Artery
PI	Pulsatility Index
PlGF	Placental Growth Factor
PRF	Pulse Repetition Frequency
RI	Resistance Index
sFlt1	Fms-like tyrosine kinase-1
TACAD	Tela-Choroidea-to-Anterior-Cerebral-Artery Distance
TAPS	Twin Anemia–Polycythemia Sequence
TI	Thermal Index
TTTS	Twin-Twin Transfusion Syndrome
UA	Umbilical Artery
UtA	Uterine Artery
VGAM	Vein of Galen Aneurysmal Malformation
VV	veno-venous
